# Association of Soyfoods or Soybean Products Consumption with Psychological Symptoms: Evidence from a Cross-Sectional Study of Chinese University Students during the COVID-19 Pandemic

**DOI:** 10.3390/ijerph20010819

**Published:** 2023-01-01

**Authors:** Shengpeng Li, Cong Liu, Yongjing Song, Nan Ma, Jinkui Lu

**Affiliations:** 1 School of Preschool Education, Jingzhou Institute of Technology, Jingzhou 434020, China; 2 Physical Education College, Jiangxi Normal University, Nanchang 330022, China; 3 College of Education and Sports Sciences, Yangtze University, Jingzhou 434020, China; 4 College of Physical Education and Health, Shanghai Lixin University of Accounting and Finance, Shanghai 201209, China; 5 School of Physical Education, Shangrao Normal University, Shangrao 334000, China

**Keywords:** soyfoods, psychological symptoms, cross-sectional analysis, university students, China

## Abstract

There is a strong association between soyfoods or soybean product consumption and adolescent health, but there are few studies on the association between soyfoods or soybean product consumption and psychological symptoms among university students. To this end, this study investigated the association between soyfoods or soybean products consumption and psychological symptoms among Chinese university students and analyzed the association between them. A three-stage stratified whole-group sampling method was used to administer questionnaires on soyfoods or soybean products consumption and psychological symptoms to 7742 university students in China. Self-assessment questionnaires were also administered to confounding variables such as basic demographic information, family status, parental education, body mass index (BMI), and moderate and vigorous physical activity (MVPA). The chi-square test, one-way ANOVA, and logistic regression analysis were used to explore the association and differences between soyfoods or soybean products consumption and psychological symptoms. The proportion of Chinese university students’ soyfoods or soybean products consumption in ≤one time/week, two–four times/week, and ≥five times/week were 38.81%, 40.24%, and 20.95%, respectively. University students’ psychological symptoms problem detection rate was 16.22%. The detection rate of psychological symptoms was lower among university male students (14.75%) than female students (17.35%), and the difference was statistically significant (χ^2^ = 9.525, *p* < 0.01). After adjusting for relevant covariates, students with soyfoods or soybean products consumption ≤one time/week (*OR* = 1.83, 95% CI:1.52, 2.21) had a higher risk of psychological symptoms compared to university students with soyfoods or soybean products consumption ≥five time/week (*p* < 0.01). During the COVID-19 pandemic, Chinese university students had lower consumption of soyfoods or soybean products and a higher detection rate of psychological symptoms. There was a negative association between soyfoods or soybean products consumption and psychological symptoms. Our study provides a scientific reference for the government and educational decision-making authorities and suggests that education on eating behavior and dietary guidance should be emphasized among university students in the future to maintain a reasonable consumption of soyfoods or soybean products for better physical and mental health development.

## 1. Introduction

As an important part of the youth population, university students have their special characteristics. They usually face the dual pressure and challenges of academic work and employment. They additionally include adaptation to a new environment when entering university [1]. Therefore, the mental health of university students deserves attention and has become a common concern in various countries [2]. Since the outbreak of the COVID-19 pandemic, university students not only have had to face the stress caused by the large amount of negative information related to the epidemic reported on social media, but also have had to face the anxiety caused by the virus and its effect on their studies and future employment. The anxiety caused by the virus has had a serious negative impact on their mental health [3]. Results of a survey of U.S. university students showed that 48.14% exhibited moderate to severe levels of depression, 38.48% exhibited moderate to severe levels of anxiety, 18.04% had suicidal thoughts, and the majority of participants (71.26%) reported that their stress/anxiety levels increased during the pandemic [4]. A survey of Chinese university students showed that 21.3% of them had anxiety problems during the COVID-19 pandemic [5]. Many factors affect the psychological symptoms of university students, such as physical activity [6], video screen behavior [7], physical fitness levels [8], sleep quality [9], and eating behavior [10]. With gradual research, it has become increasingly clear that diet has an impact on mental health. Studies have confirmed that diet is as important to psychiatry as it is to cardiology, endocrinology, and gastroenterology, thus indicating that eating behaviors have an important impact on mental health [11].

It is well known that changes in dietary behavior are a global trend that may be related to industrialization and economic development [12]. Soy products have played an important role in the cooking of many Asian countries for centuries. Soy is a good source of protein, dietary fiber, and several phytochemicals such as isoflavones, phytic acid, trypsin inhibitors, and saponins [13]. Soy products have many health benefits, including the prevention of coronary heart disease, breast and prostate cancer, osteoporosis, and the relief of menopausal symptoms [14,15]. Similarly, this significant association between soy product consumption and all-cause mortality exists [16]. Research on the effects of soy products on human health has been conducted over the past 30 years. In addition to their antioxidant effects, soy products have other bioactive properties such as immunomodulation, anticancer activity, prevention of cardiovascular disease, Alzheimer’s disease prevention, and cholesterol reduction [17,18,19,20]. The COVID-19 pandemic has affected people’s dietary habits, which may include changes in the consumption of soy products; therefore, the study of soy product consumption in the dietary behavior of university students deserves attention.

The availability and consumption of soyfoods or soybean products have increased significantly in the United States as well as in many developed countries in the past decades. In many countries, in addition to the traditional Asian soyfoods or soybean products, whole soybean or soybean protein products such as isolated soy protein (ISP) and soy protein concentrate are available [21]. With the development of soyfoods or soybean products, increasing attention has been paid abroad to the positive effects of soyfoods or soybean products on health, including mental health. A study on young people in the United States found that eating foods rich in high-quality soy protein was beneficial for certain aspects of mood and cognition, and the effects were positive [22]. Several studies have also revealed the health benefits of soy products, including antioxidants, and even blood pressure-lowering effects [23]. In addition, a Chinese study found that soyfoods or soybean product intake was negatively associated with the risk of depression [24]. However, the Chinese study was conducted on a rural sample population aged 35 years or older. In contrast, fewer studies have been conducted on the Chinese university student population regarding soyfoods or soybean product consumption and psychological symptoms [24].

Given the important proportion of the university students group in the total population of the country and the importance it plays in the future economic development of the country, it is necessary to investigate the eating behavior and psychological symptoms of this group. Coupled with the impact of the COVID-19 pandemic in recent years, this has further deepened the psychological burden of the university students group. Therefore, this study investigated the consumption of soyfoods or soybean products and psychological symptoms among 7742 university students in China to analyze the association that exists between the two. It provides an important reference for Chinese governmental departments and educational policymakers, as well as a reference that helps to promote the development and intervention of eating behaviors and physical and mental health of university students in China.

## 2. Materials and Methods

### 2.1. Participants

**Figure 1:**
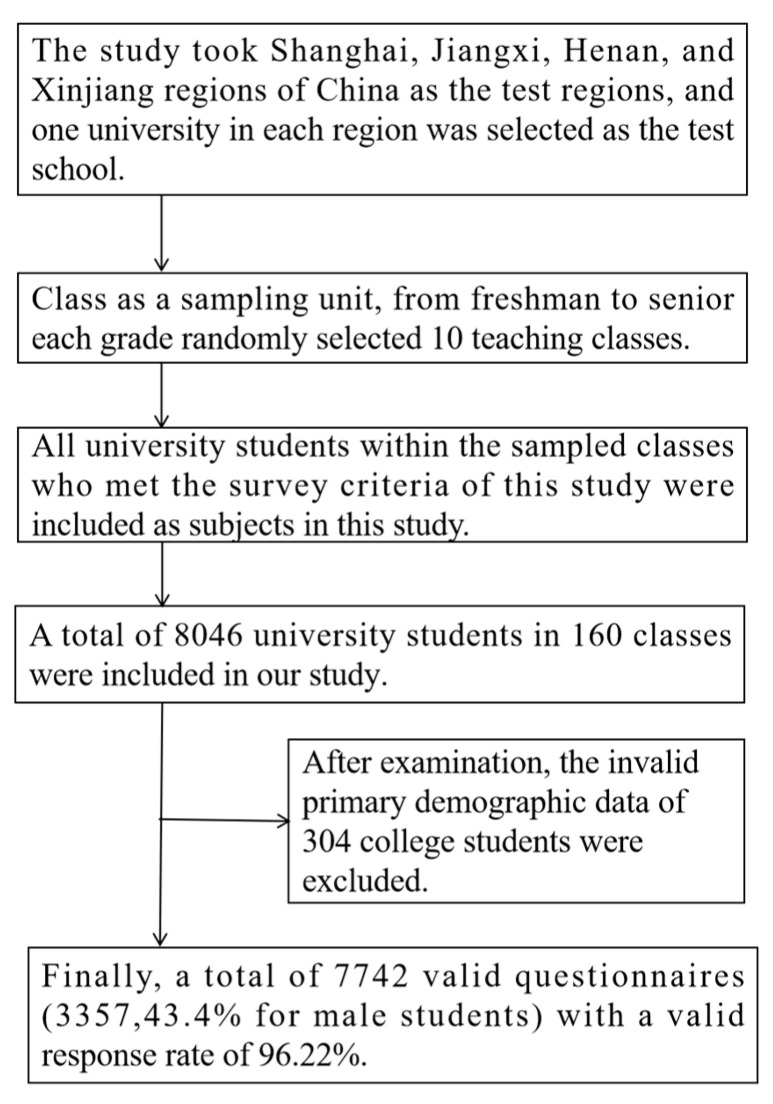
University students’ subject extraction process.

Our study was conducted using a stratified whole-group sampling method, and the process of drawing subjects was divided into three steps. First, considering the geographical distribution of Chinese provinces from east to west and north to south, our study took the Shanghai, Jiangxi, Henan, and Xinjiang regions of China as the test regions, and one university in each region was selected as the test school. In the second step, at each university, 10 teaching classes were randomly selected in each grade from freshman to senior year, and we used classes as the sampling unit. In the third step, all university students within the sampled classes who met the survey criteria of this study were included as subjects in the study. A total of 8046 university students in 160 classes were included in our study, and a total of 7742 valid questionnaires (3357, 43.4% for male students) were returned following the exclusion of 304 university students who presented invalid primary demographic information after the survey test, with a valid response rate of 96.22%. The specific sampling process for the subjects in this study is shown in Figure 1.

This research investigation was approved by the Human Ethics Committee of Shangrao Normal University (202105012). Written informed consent was obtained from the university students themselves before the survey. To strictly protect the privacy of the students, the questionnaire was completed using anonymous numbering for the survey.

### 2.2. Data Collection Process

An electronic questionnaire was used in our study. The survey staff consisted of faculty and graduate students who had been trained and then tested. The staff used uniform instructional language to explain the purpose and requirements of the survey to the subjects before the survey. Students scanned the QR code on the spot to fill in the electronic questionnaire and submitted the questionnaire on the spot after completion. The staff were responsible for answering any questions in the process of filling out the questionnaire, and the staff were present during the whole process of filling out the questionnaire. The questionnaire contained basic information on soyfoods or soybean products consumption, psychological symptoms, etc.

### 2.3. Soyfoods or Soybean Products Consumption

The soyfoods or soybean products consumption data were obtained through a questionnaire survey of the subjects with the following questions. How many times did you consume soyfoods or soybean products in the past week, such as tofu, dried tofu, bean curd, soybean products, etc.? To ensure the adequacy of participants, our study divided soyfoods or soybean products consumption into three groups for the study, ≤one time/week, two–four times/week, and ≥five times/week, respectively.

### 2.4. Psychological Symptoms

Our study was conducted using a questionnaire suitable for Chinese children and adolescents’ psychological symptoms developed by the team of the Chinese scholar Professor Tao Fangbiao, which has been used in several studies [25,26]. The multidimensional sub-health questionnaire of adolescents (MSQA) is a psychological symptoms questionnaire designed for a group of Chinese children and adolescents. The MSQA short form with good reliability and validity was used to survey in this study. The scale consists of 15 items, divided into three dimensions: emotional symptoms, behavioral symptoms, and social adaptation difficulties, and the scores of each dimension are summed up to obtain the total psychological symptoms score. The emotional symptoms consisted of seven items, such as “often feel nervous”, etc. The behavioral symptoms consisted of four items. Behavioral symptoms consisted of four items, such as “often argues with others”, etc. The social adaptation difficulties consisted of four items, such as “not feeling energized about anything all day long”. Each item was selected according to the participant’s actual situation in the past six months, and was divided into “lasting more than 3 months”, “lasting more than 2 months”, “lasting more than 1 month”, and “lasting more than 2 weeks”. The numbers “more than 2 weeks”, “more than 1 week”, and “none or less than 1 week” are 1–6, respectively. A score of 1 was recorded when serial numbers 1–3 were selected, and a score of 0 was recorded when serial numbers 4–6 were selected. A score of ≥4 for emotional symptoms, ≥1 for behavioral symptoms, and ≥2 for social adaptation difficulties indicated a positive result for this dimension. The total score of the questionnaire was 0–15, and a total score of ≥7 was determined as the presence of mental health problems in the subjects.

### 2.5. Covariates

The investigation of covariates in our study included age, sex, being an only child, father’s education, mother’s education, socioeconomic status (SES), body mass index (BMI), and moderate and vigorous physical activity (MVPA). Age was calculated in weeks, e.g., 20 years old means 20.0–20.99 years old. The only child was divided into two categories, Yes and No. Father’s education was divided into three categories, namely, primary school or below, secondary and high school, and junior college or above. Mother’s education was divided into three categories, namely, primary school or below, secondary and high school, and junior college or above. SES was divided into three categories: low (<25th percentile), medium (25–75th percentile), and high (>75th percentile). The BMI was calculated based on weight (kg)/height (m)2. Height and weight were tested according to the testing methods and instruments required by the China National Student Physical Fitness Survey, and height was accurate to 0.1 cm and weight was accurate to 0.1 kg [27]. MVPA was investigated using the survey entries in the physical activity section of the China National Student Physical Health Survey questionnaire. The average daily frequency and duration of moderate and vigorous physical activity in the past seven days were investigated to calculate the daily MVPA time of the subjects. The medium- to high-intensity physical activities included fast running, ball games, heavy lifting, ice skating, skiing, and cycling.

### 2.6. Statistical Analysis

Categorical variables for different categories of Chinese university students’ soyfoods or soybean products consumption were presented as percentages (*N*, %), and continuous variables were expressed as mean and standard deviation (M ± SD). Comparisons between different categorical variables were conducted using chi-square tests. Comparisons of continuous variables were performed by one-way ANOVA. Comparisons between different soyfoods or soybean products consumption, university students’ emotional symptoms, behavioral symptoms, social adaptation difficulties, and psychological symptoms were examined by a chi-square test. The association between university students’ soyfoods or soybean products consumption and psychological symptoms was analyzed by logistic regression. The presence or absence of psychological symptoms in university students was used as the dependent variable, and soy products were used as the independent variable. The association of university students’ soyfoods or soybean products consumption with emotional symptoms, behavioral symptoms, social adaptation difficulties, and psychological symptoms was analyzed using a crude model, Model 1 and Model 2, respectively, reporting the odds ratio and a 95% confidence interval. Model 1 adjusted for age, being an only child, parental education, and SES; Model 2 adjusted for BMI and MVPA on the basis of Model 1. Data were processed using SPSS25.0 (SPSS Inc., Chicago, IL, USA) software. A two-sided test level of α = 0.05 was used.

## 3. Results

Our study was a cross-sectional retrospective study that examined the consumption of soyfoods or soybean products among 7742 Chinese university students, which was conducted during the COVID-19 pandemic. The mean age of the subjects in this study was (20.16 ± 1.03) years, of whom 3357 (43.4%) were male students. The results showed that the proportion of Chinese university students’ soyfoods or soybean products consumption in ≤one time/week, two–four times/week, and ≥five times/week were 38.81%, 40.24%, and 20.95%, respectively.

In terms of count variables, our study showed that the differences in the detection rates of soyfoods or soybean products consumption among Chinese university students compared across sex, being an only child, father’s education, mother’s education, and SES were all statistically significant (χ^2^ values 24.077, 18.064, 65.815, 47.362, 70.893, *p* < 0.001). For continuous variables, there was a difference in BMI among Chinese university boys with different consumption of soy foods or soy products (*F* value = 3.788. *p* < 0.05). There was a statistically significant difference in MVPA between boys and girls among Chinese university students who consumed different soy foods or soy products (*F* value = 30.115, 18.764. *p* < 0.001).

Overall, a higher proportion of college students who were female students, non-only children, had fathers with secondary and high school education, had mothers with secondary and high school education, and moderate SES consumed soy foods or soy products ≥five times/week. University boys and girls who consumed soy products ≥five times/week had longer MVPA of (31.15 ± 24.98) min/day and (20.47 ± 15.77) min/day, respectively (Table 1).

Our survey showed that the detection rate of psychological symptoms among Chinese university students during the COVID-19 pandemic was 16.22% (1256/7742). Among them, the detection rate of psychological symptoms was 14.75% (495/3357) in male students and 17.35% (761/4385) in female students. The difference in the detection rate of psychological symptoms between genders was statistically significant (χ^2^ = 9.525, *p* < 0.01).

Overall, university students who consumed soy foods or soy products ≤one time/week had a 20. 0% detection rate of psychological symptoms, which was higher than those who consumed soy foods or soy products two–four times/week (15.4%) and ≥five times/week (10.7%), with a statistically significant difference (χ^2^ = 70.354, *p* < 0.001). Regarding the different dimensions, university students who consumed soy foods or soy products ≤one time/week had the highest detection rates of emotional symptoms, behavioral symptoms, and social adjustment difficulties, 21.4%, 21.0%, and 17.7%, respectively, which were higher than those who consumed soyfoods or soy products two–four times/week and ≥five times/week, and the differences were statistically significant (χ^2^ = 58.593, 41.502, 43.655, *p* < 0.001). The same trend was found between the different genders (Table 2).

After the relevant covariates had been adjusted, Model 2 analysis results showed that compared with university students with soyfoods or soybean products consumption ≥five times/week, university students with soyfoods or soybean products consumption ≤one time/week (*OR* = 1.83, 95% CI: 1.52, 2.21) were at higher risk of developing psychological symptoms (*p* < 0.01). Similarly, compared to university students with soyfoods or soybean products consumption ≥five times/week, soyfoods or soybean products consumption emotional symptoms in university students ≤one time/week (*OR* = 1.58, 95% CI: 1.33, 1.89), behavioral symptoms (*OR* = 1.46, 95% CI: 1.23, 1.73), social adaptation difficulties (*OR* = 1.48, 95% CI: 1.23, 1.77). The risk was also higher (*p* < 0.01). Males and females showed the same trend in general. Our results suggest a negative association between university students’ soyfoods or soybean products consumption and the occurrence of psychological symptoms in China during the COVID-19 pandemic (Table 3).

## 4. Discussion

China is the homeland of soybeans, and soyfoods or soybean products have been cultivated in China for 5000 years and have had a profound impact on the dietary behavior of Chinese people [28]. Our results showed that the proportion of Chinese university students’ soyfoods or soybean products consumption ≥five times/week was only 20.95%; however, 38.81% of university students’ soyfoods or soybean products consumption ≤one time/week. This result indicates that the overall proportion of Chinese university students’ soyfoods or soybean products consumption is low, which is consistent with the analysis results of the survey in the China 2021 Dietary Guidelines. The proportion of soyfoods or soybean products currently being consumed in China is low, and about 40% of adults do not eat soyfoods or soybean products regularly [29]. However, Chinese university students’ soyfoods or soybean products consumption is greater than that of university students in European and American countries. Studies have shown that only 4.5% of American respondents aged 9–18 years reported consuming soyfoods or soybean products [30]. Only 2.8% of Canadian respondents aged 2–18 years reported soyfoods or soybean product consumption [31]. The disparity in soyfoods or soybean product consumption between Chinese and Western populations is strongly associated with differences in traditional dietary practices.

Our study also showed that the detection rate of psychological symptoms among Chinese university students was 16.22%. This result was low compared to other studies [32]. A mental health meta-analysis of adolescents during the COVID-19 pandemic showed a 41.7% detection rate of depressive symptoms [33]. The prevalence of psychiatric problems among Italian adolescents during the COVID-19 pandemic was 38.5% [34]. Studies have shown that the detection rate of psychological symptoms among Chinese university students has revealed a decreasing trend year by year, and the incidence of problems including suicidal behavior, depression, and anxiety has been decreasing, which is closely related to the emphasis on mental health education for university students in recent years [35]. In addition, the continued decline in the detection rate of psychological symptoms among university students can be explained by the rapid growth of the Chinese economy over the past 40 years. With the increase in income and improvement in daily life, Chinese people, including Chinese university students, have become the direct beneficiaries of wealth. As a result, they are more optimistic about their lives and the future of their infrastructure, and the increased optimism and life satisfaction reduce the stress caused by unfulfilled aspirations and therefore reduce the risk of psychological symptoms [36]. However, our study was carried out during the COVID-19 pandemic and remains higher compared to previous findings in Chinese university students (14.2%) [37]. The increase in psychological symptoms was associated with limited socialization, decreased physical activity, reduced recreational activities, and altered eating behaviors associated with the COVID-19 pandemic [38,39]. Our study also showed that the detection rate of psychological symptom problems in Chinese university students was higher in female students (17.35%) than in male students (14.75%). Several studies have confirmed [40,41] that female students are more introverted by nature and are less likely to confide in others when they encounter problems. On the contrary, boys are more extroverted and are used to releasing their problems through confiding, games, and sports, which leads to this result.

Several studies have shown that different eating behaviors and types of diets are associated with psychological symptoms. In particular, the association between the intake of red meat [42], dairy products [43], and sugary foods [44] and psychological symptoms has been studied more frequently, while fewer studies have been conducted at home and abroad on the association between the consumption of soyfoods or soybean products and psychological symptoms in adolescents. With the continuous improvement in living standards, studies on the association between psychological symptoms and soyfoods or soybean product consumption have been increasing and attracting attention in recent years. In our study, we found a negative association between soyfoods or soybean products consumption and psychological symptoms in university students, i.e., those with higher consumption of soyfoods or soybean products had lower detection rates of psychological symptoms. In a cross-sectional study of elderly residents in rural northeastern China [45], soyfoods or soybean products consumption was negatively associated with the risk of depression, i.e., individuals who consumed soyfoods or soybean products ≥four times per week were significantly less likely to develop depressive symptoms than those who rarely consumed soyfoods or soybean products. A small cross-sectional study in Japan involving 89 pre- and postmenopausal females found a significant negative association between soyfoods or soybean products consumption and the onset of depression [46]. However, the results were not entirely consistent. A cohort study of 1609 adults in Taiwan, China, showed no significant association between soyfoods or soybean products consumption and the occurrence of depression [47]. The reasons for the differences in the results of different studies are related to some differences in the criteria used to investigate soyfoods or soybean product consumption in different studies.

The association that exists between soyfoods or soybean products consumption and psychological symptoms can be analyzed in terms of the secretion of brain hormones by the nutritional composition of soyfoods or soybean products. Emerging evidence that isoflavones in soy have antidepressant effects is of particular interest [48]. Animal experiments have revealed potential mechanisms of action of isoflavones, and soy isoflavones may influence monoamine neurotransmitters by reshaping the structure of the intestinal flora, thereby alleviating depression [49]. In addition, soy isoflavones, cis-PUFAs, and high-quality plant proteins all improve the cognitive performance of the brain [50]. Soy isoflavones may affect brain function through ER-mediated processes and inhibition of tyrosine kinases and may prevent neuronal damage and cognitive decline [51]. It has also been found that soy protein promotes an increase in muscle strength [52]. Additionally, a growing body of evidence suggests a strong association between muscle strength and mental health [53,54]. In addition to protein and isoflavones, soy has many other functions and nutrient components such as fatty acids, vitamins, peptides, minerals, flavonoids, etc. [23]. However, some studies confirm that multivitamins also make a positive contribution to mental health [55]. In summary, soyfoods or soybean products tend to reduce confusion and increase cognitive flexibility of the brain in those who consume them, and they may prevent a decline in feelings of vitality and enhance feelings of pleasure, thus promoting psychological well-being and reducing the occurrence of psychological symptoms [56].

Our study has certain advantages. First, to the best of our knowledge, this study is the first to examine the correlation between soyfoods or soybean products and psychological symptoms in Chinese university students. It can provide a reference for the later dietary behavior and mental health interventions of university students. Secondly, this study has a wide survey area, a certain sample, and a good representation. However, there are some limitations to our study. First, this was a cross-sectional survey study, and it was not possible to analyze the causal association between soyfoods or soybean product consumption and psychological symptoms. Second, this was a self-reported retrospective survey that required subjects to recall their eating behaviors and psychological symptoms in the past. Some discrepancies with the true situation are inevitable due to individual recall ability.

## 5. Conclusions

Our study confirmed that Chinese university students’ soyfoods or soybean products consumption was lower, and the psychological symptoms detection rate was higher, during the COVID-19 pandemic. There was a negative association between soyfoods or soybean product consumption and psychological symptoms. The results of our study provide a strong reference for governmental and educational decision-makers to focus on dietary behavior education and dietary guidance among university students to maintain reasonable soyfoods or soybean product consumption in the future. In addition, the results of this study suggest that we need to strengthen mental health education and guidance for Chinese university students during the COVID-19 pandemic to reduce the occurrence of psychological symptoms among university students and to better promote physical and mental health development.

## Figures and Tables

**Table 1:** Comparison of the consumption status of soy food or soy products among college students with different characteristics in China.

Characteristics	Soyfoods or Soybean Products Consumption			Total Sample	*χ*^2^/*F*-Value	*p*-Value
	≤1 *t*/*w*	2–4 *t*/*w*	≥5 *t*/*w*			
*N*	3005	3115	1622	7742		
Age (years)	20.21 ± 1.05	20.15 ± 1.04	20.06 ± 0.98	20.16 ± 1.03	10.632	<0.001
Sex						
Boys	1205 (40.1)	1388 (44.6)	764 (47.1)	3357 (43.4)	24.077	<0.001
Girls	1800 (59.9)	1727 (55.4)	858 (52.9)	4385 (56.6)		
Only child						
Yes	695 (23.1)	818 (26.3)	464 (28.6)	1977 (25.5)	18.064	<0.001
No	2310 (76.9)	2297 (73.7)	1158 (71.4)	5765 (74.5)		
Father’s education						
Primary School or below	981 (32.6)	840 (27)	372 (22.9)	2193 (28.3)	65.815	<0.001
Secondary and high school	1825 (60.7)	2017 (64.8)	1078 (66.5)	4920 (63.5)		
Junior college or above	199 (6.6)	258 (8.3)	172 (10.6)	629 (8.1)		
Mother’s education						
Primary School or below	1500 (49.9)	1408 (45.2)	653 (40.3)	3561 (46.0)	47.362	<0.001
Secondary and high school	1390 (46.3)	1560 (50.1)	867 (53.5)	3817 (49.3)		
Junior college or above	115 (3.8)	147 (4.7)	102 (6.3)	364 (4.7)		
SES						
Low	572 (19)	452 (14.5)	183 (11.3)	1207 (15.6)	70.893	<0.001
Medium	2083 (69.3)	2238 (71.8)	1158 (71.4)	5479 (70.8)		
High	350 (11.6)	425 (13.6)	281 (17.3)	1056 (13.6)		
BMI (kg/m^2^)						
Boys	24.28 ± 5.85	23.66 ± 5.76	23.84 ± 5.86	23.93 ± 5.82	3.788	0.023
Girls	23.18 ± 6.69	22.67 ± 6.35	22.78 ± 6.77	22.90 ± 6.58	2.876	0.056
MVPA (min/day)						
Boys	28.10 ± 19.87	24.00 ± 20.01	31.15 ± 24.98	27.10 ± 21.38	30.115	<0.001
Girls	18.08 ± 14.05	16.86 ± 13.28	20.47 ± 15.77	18.07 ± 14.17	18.764	<0.001

Note: Descriptive statistics are presented as mean (standard deviation) and number (percentage) for continuous and categorical. Socioeconomic status(SES)Low(<25th SES), Medium(25–75th SES), High(>75th SES). Abbreviations: *t*/*w*, *time*/*week*; BMI, body mass index; MVPA, moderate and vigorous physical activity.

**Table 2:** Comparison of detection rates of psychological symptoms among university students consuming different soyfoods or soybean products in China.

Psychological Symptoms	Soyfoods or Soybean Products Consumption	*N*	Percentage (%)	*Χ*^2^-Value	*p*-Value
Boys					
Emotional symptoms	≤1 *t*/*w*	246	20.4	29.708	<0.001
	2–4 *t*/*w*	180	13.0		
	≥5 *t*/*w*	106	13.9		
Behavioral symptoms	≤1 *t*/*w*	239	19.8	17.115	<0.001
	2–4 *t*/*w*	196	14.1		
	≥5 *t*/*w*	113	14.8		
Social adaptation difficulties	≤1 *t*/*w*	232	19.3	37.416	<0.001
	2–4 *t*/*w*	175	12.6		
	≥5 *t*/*w*	78	10.2		
Psychological symptoms	≤1 *t*/*w*	233	19.3	33.133	<0.001
	2–4 *t*/*w*	179	12.9		
	≥5 *t*/*w*	83	10.9		
Girls					
Emotional symptoms	≤1 *t*/*w*	397	22.1	37.547	<0.001
	2–4 *t*/*w*	324	18.8		
	≥5 *t*/*w*	104	12.1		
Behavioral symptoms	≤1 *t*/*w*	393	21.8	30.711	<0.001
	2–4 *t*/*w*	329	19.1		
	≥5 *t*/*w*	110	12.8		
Social adaptation difficulties	≤1 *t*/*w*	299	16.6	11.982	0.003
	2–4 *t*/*w*	230	13.3		
	≥5 *t*/*w*	105	12.2		
Psychological symptoms	≤1 *t*/*w*	369	20.5	40.631	<0.001
	2–4 *t*/*w*	302	17.5		
	≥5 *t*/*w*	90	10.5		
Total					
Emotional symptoms	≤1 *t*/*w*	643	21.4	58.593	<0.001
	2–4 *t*/*w*	504	16.2		
	≥5 *t*/*w*	210	12.9		
Behavioral symptoms	≤1 *t*/*w*	632	21.0	41.502	<0.001
	2–4 *t*/*w*	525	16.9		
	≥5 *t*/*w*	223	13.7		
Social adaptation difficulties	≤1 *t*/*w*	531	17.7	43.655	<0.001
	2–4 *t*/*w*	405	13.0		
	≥5 *t*/*w*	183	11.3		
Psychological symptoms	≤1 *t*/*w*	602	20.0	70.354	<0.001
	2–4 *t*/*w*	481	15.4		
	≥5 *t*/*w*	173	10.7		

Note: *t*/*w*, *time*/*week*.

**Table 3:** Logistic regression analysis of university students’ soyfoods or soybean products consumption and psychological symptoms in China (*n* = 7742).

Psychological Symptoms	Soyfoods or Soybean Products Consumption	Odds Ratio (95% Confidence Interval)		
		Crude Model	Model 1	Model 2
Boys				
Emotional symptoms	≥5 *t*/*w*	1.00 (Reference)	1.00 (Reference)	1.00 (Reference)
	2–4 *t*/*w*	0.93 (0.72, 1.2)	0.88 (0.67, 1.14)	0.78 (0.6, 1.02)
	≤1 *t*/*w*	1.59 (1.24, 2.04) ^a^	1.45 (1.13, 1.87) ^a^	1.39 (1.08, 1.8) ^a^
	*p* for trend	<0.001	<0.001	<0.001
Behavioral symptoms	≥5 *t*/*w*	1.00 (Reference)	1.00 (Reference)	1.00 (Reference)
	2–4 *t*/*w*	0.95 (0.74, 1.22)	0.91 (0.71, 1.17)	0.85 (0.66, 1.11)
	≤1 *t*/*w*	1.43 (1.12, 1.82)	1.32 (1.03, 1.69)	1.27 (0.99, 1.63)
	*p* for trend	<0.001	<0.001	<0.001
Social adaptation difficulties	≥5 *t*/*w*	1.00 (Reference)	1.00 (Reference)	1.00 (Reference)
	2–4 *t*/*w*	1.27 (0.96, 1.68)	1.21 (0.91, 1.61)	1.15 (0.86, 1.53)
	≤1 *t*/*w*	2.1 (1.59, 2.76) ^a^	1.93 (1.46, 2.55) ^a^	1.88 (1.42, 2.49) ^a^
	*p* for trend	<0.001	<0.001	<0.001
Psychological symptoms	≥5 *t*/*w*	1.00 (Reference)	1.00 (Reference)	1.00 (Reference)
	2–4 *t*/*w*	1.22 (0.92, 1.6)	1.16 (0.88, 1.54)	1.03 (0.78, 1.37)
	≤1 *t*/*w*	1.97 (1.5, 2.57) ^a^	1.8 (1.37, 2.37) ^a^	1.75 (1.32, 2.31) ^a^
	*p* for trend	<0.001	<0.001	<0.001
Girls				
Emotional symptoms	≥5 *t*/*w*	1.00 (Reference)	1.00 (Reference)	1.00 (Reference)
	2–4 *t*/*w*	1.67 (1.32, 2.12) ^a^	1.6 (1.26, 2.04) ^a^	1.43 (1.12, 1.84)
	≤1 *t*/*w*	2.05 (1.63, 2.59) ^a^	1.87 (1.47, 2.37) ^a^	1.76 (1.38, 2.26) ^a^
	*p* for trend	<0.001	<0.001	<0.001
Behavioral symptoms	≥5 *t*/*w*	1.00 (Reference)	1.00 (Reference)	1.00 (Reference)
	2–4 *t*/*w*	1.6 (1.27, 2.02) ^a^	1.54 (1.21, 1.94) ^a^	1.39 (1.09, 1.77)
	≤1 *t*/*w*	1.9 (1.51, 2.39) ^a^	1.73 (1.37, 2.19) ^a^	1.64 (1.29, 2.08) ^a^
	*p* for trend	<0.001	<0.001	<0.001
Social adaptation difficulties	≥5 *t*/*w*	1.00 (Reference)	1.00 (Reference)	1.00 (Reference)
	2–4 *t*/*w*	1.1 (0.86, 1.41)	1.04 (0.81, 1.34)	0.99 (0.77, 1.28)
	≤1 *t*/*w*	1.43 (1.13, 1.81) ^a^	1.29 (1.01, 1.64)	1.22 (0.96, 1.56)
	*p* for trend	<0.001	<0.001	<0.001
Psychological symptoms	≥5 *t*/*w*	1.00 (Reference)	1.00 (Reference)	1.00 (Reference)
	2–4 *t*/*w*	1.81 (1.41, 2.33) ^a^	1.73 (1.34, 2.23) ^a^	1.54 (1.19, 2) ^a^
	≤1 *t*/*w*	2.2 (1.72, 2.82) ^a^	2 (1.56, 2.57) ^a^	1.91 (1.47, 2.47) ^a^
	*p* for trend	<0.001	<0.001	<0.001
Total				
Emotional symptoms	≥5 *t*/*w*	1.00 (Reference)	1.00 (Reference)	1.00 (Reference)
	2–4 *t*/*w*	1.3 (1.09, 1.54) ^a^	1.24 (1.04, 1.48)	1.1 (0.92, 1.31)
	≤1 *t*/*w*	1.83 (1.55, 2.17) ^a^	1.68 (1.41, 1.99) ^a^	1.58 (1.33, 1.89) ^a^
	*p* for trend	<0.001	<0.001	<0.001
Behavioral symptoms	≥5 *t*/*w*	1.00 (Reference)	1.00 (Reference)	1.00 (Reference)
	2–4 *t*/*w*	1.27 (1.07, 1.51)	1.22 (1.03, 1.45)	1.11 (0.93, 1.32)
	≤1 *t*/*w*	1.67 (1.42, 1.97) ^a^	1.54 (1.3, 1.83) ^a^	1.46 (1.23, 1.73) ^a^
	*p* for trend	<0.001	<0.001	<0.001
Social adaptation difficulties	≥5 *t*/*w*	1.00 (Reference)	1.00 (Reference)	1.00 (Reference)
	2–4 *t*/*w*	1.18 (0.98, 1.42)	1.12 (0.93, 1.35)	1.06 (0.88, 1.29)
	≤1 *t*/*w*	1.69 (1.41, 2.02) ^a^	1.54 (1.28, 1.85) ^a^	1.48 (1.23, 1.77) ^a^
	*p* for trend	<0.001	<0.001	<0.001
Psychological symptoms	≥5 *t*/*w*	1.00 (Reference)	1.00 (Reference)	1.00 (Reference)
	2–4 *t*/*w*	1.53 (1.27, 1.84) ^a^	1.46 (1.21, 1.76) ^a^	1.29 (1.06, 1.56)
	≤1 *t*/*w*	2.1 (1.75, 2.52) ^a^	1.92 (1.6, 2.31) ^a^	1.83 (1.52, 2.21) ^a^
	*p* for trend	<0.001	<0.001	<0.001

Note: Model 1 adjusted for age, an only child, parental education, and SES; Model 2 adjusted for BMI and MVPA based on Model 1. ^a^, *p* < 0.01.

## Data Availability

To protect the privacy of participants, the questionnaire data will not be disclosed to the public. If necessary, you can contact the corresponding author.
